# Clinical outcomes using carbon-ion radiotherapy and dose-volume histogram comparison between carbon-ion radiotherapy and photon therapy for T2b-4N0M0 non-small cell lung cancer—A pilot study

**DOI:** 10.1371/journal.pone.0175589

**Published:** 2017-04-11

**Authors:** Katsuyuki Shirai, Motohiro Kawashima, Jun-ichi Saitoh, Takanori Abe, Kyohei Fukata, Yuka Shigeta, Daisuke Irie, Shintaro Shiba, Naoko Okano, Tatsuya Ohno, Takashi Nakano

**Affiliations:** Gunma University Heavy Ion Medical Center, Maebashi, Gunma, Japan; ENEA Centro Ricerche Casaccia, ITALY

## Abstract

The safety and efficacy of carbon-ion radiotherapy for advanced non-small cell lung cancer have not been established. We evaluated the clinical outcomes and dose-volume histogram parameters of carbon-ion radiotherapy compared with photon therapy in T2b–4N0M0 non-small cell lung cancer. Twenty-three patients were treated with carbon-ion radiotherapy between May 2011 and December 2015. Seven, 14, and 2 patients had T2b, T3, and T4, respectively. The median age was 78 (range, 53−91) years, with 22 male patients. There were 12 adenocarcinomas, 8 squamous cell carcinomas, 1 non-small cell lung carcinoma, and 2 clinically diagnosed lung cancers. Eleven patients were operable, and 12 patients were inoperable. Most patients (91%) were treated with carbon-ion radiotherapy of 60.0 Gy relative biological effectiveness (RBE) in 4 fractions or 64.0 Gy (RBE) in 16 fractions. Local control and overall survival rates were calculated. Dose-volume histogram parameters of normal lung and tumor coverages were compared between carbon-ion radiotherapy and photon therapies, including three-dimensional conformal radiotherapy (3DCRT) and intensity-modulated radiotherapy (IMRT). The median follow-up of surviving patients was 25 months. Three patients experienced local recurrence, and the 2-year local control rate was 81%. During follow-up, 5 patients died of lung cancer, and 1 died of intercurrent disease. The 2-year overall survival rate was 70%. Operable patients had a better overall survival rate compared with inoperable patients (100% *vs*. 43%; *P* = 0.04). There was no grade ≥2 radiation pneumonitis. In dose-volume histogram analysis, carbon-ion radiotherapy had a significantly lower dose to normal lung and greater tumor coverage compared with photon therapies. Carbon-ion radiotherapy was effectively and safely performed for T2b–4N0M0 non-small cell lung cancer, and the dose distribution was superior compared with those for photon therapies. A Japanese multi-institutional study is ongoing to prospectively evaluate these patients and establish the use of carbon-ion radiotherapy.

## Introduction

Lung cancer is the leading cause of morbidity and mortality worldwide. Surgical resection is the standard treatment for non-small lung cancer (NSCLC) without distant metastasis [1]. However, lung cancer is a disease of the elderly, and it is difficult to treat inoperable patients with locally advanced NSCLC [2]. For unresectable stage IIIA disease with mediastinal lymph node metastasis (N2), concurrent chemoradiotherapy has been reported to improve overall survival (OS) rates compared with radiotherapy alone [3–5]. However, treatment for inoperable locally advanced NSCLC without lymph node metastasis has not been established, and the clinical outcomes with radiotherapy alone are unsatisfactory [6].

Carbon-ion radiotherapy has good dose-localizing properties because of the Bragg peak, and the dose to the surrounding normal tissue can be minimized [7]. Furthermore, a carbon-ion beam offers high biological effectiveness, which results in favorable tumor control. Therefore, carbon-ion radiotherapy is considered a radical non-surgical therapy for achieving high local control rates without severe adverse events [8, 9]. Recently, a few studies on carbon-ion radiotherapy for stage I NSCLC have been reported, and the results were comparable to those for stereotactic body radiotherapy (SBRT) [10–13]. However, the safety and efficacy of carbon-ion radiotherapy for T2b–4N0M0 NSCLC have not been established. In the present study, we evaluated the clinical outcomes and dose-volume histogram (DVH) parameters of carbon-ion radiotherapy compared with photon therapy for T2b–4N0M0 NSCLC.

## Materials and methods

### Patient and tumor characteristics

Between May 2011 and December 2015, 23 patients with T2b−T4N0M0 NSCLC were treated with carbon-ion radiotherapy at Gunma University Hospital (Gunma, Japan). The present study did not enroll patients with lymph node metastasis because they were being accrued for another prospective study (UMIN000011041). All patients provided written informed consent before commencing treatment. This retrospective study was approved (No. 160030) by Gunma University Ethical Review Board for Medical Research Involving Human Subjects and was conducted in accordance with the Declaration of Helsinki. Data are available from the Gunma University Heavy Ion Medical Center and the Ethical Review Board for researchers who meet the criteria for access to confidential data (GHMC@ml.gunma-u.ac.jp).

A summary of the patient and tumor characteristics is provided in **Table 1**. The median age was 78 (range, 53–91) years. Twenty-two patients (96%) were male, and 1 (4%) was female. The cohort comprised 12 adenocarcinomas, 8 squamous cell carcinomas, 1 non-small cell carcinoma, and 2 clinically diagnosed lung cancers. Most patients (96%) had a good performance status (PS) of 0–1 and 1 (4%) had a poor PS due to severe pulmonary dysfunction. Half of those with a good PS were medically inoperable because of impaired pulmonary function (n = 4), vertebral body invasion (n = 2), old age (n = 2), severe cardiac complications (n = 2), and dementia (n = 1). In addition, although considered to have operable tumors, some patients (n = 11) refused surgery in the hope of undergoing carbon-ion radiotherapy. Seven, 14, and 2 patients had T2b, T3, and T4, respectively. The median tumor length was 62 (range, 26−95) mm.

**Table 1:** Patient and tumor characteristics.

All patients		n = 23	(100%)
Age	Median (years)	78	(range, 53−91)
Sex	Male	22	(96%)
	Female	1	(4%)
Performance status	0	2	(9%)
	1	20	(87%)
	2	1	(4%)
Operability	Operable	11	(48%)
	Inoperable	12	(52%)
Histology	Adenocarcinoma	12	(52%)
	Squamous cell carcinoma	8	(35%)
	Non-small cell lung carcinoma	1	(4%)
	Clinically diagnosed lung cancer	2	(9%)
T stage	T2b	7	(30%)
	T3	14	(61%)
	T4	2	(9%)
Tumor length	Median (mm)	62	(range, 26−95)
GTV	Median (cm^3^)	59	(range, 11−371)
Radiation dose	52.8 Gy (RBE) / 4 fractions	1	(4%)
	60.0 Gy (RBE) / 4 fractions	14	(61%)
	64.0 Gy (RBE) / 16 fractions	7	(30%)
	70.4 Gy (RBE) / 16 fractions	1	(4%)

RBE, relative biological effectiveness; GTV, Gross tumor volume.

### Treatment planning

Details of the carbon-ion radiotherapy and treatment planning techniques used in this study have been reported elsewhere [13, 14]. Briefly, carbon-ion beams (290–400 MeV) were generated using a heavy particle accelerator at Gunma University Heavy Ion Medical Center (Gunma, Japan). Patients were immobilized on fixation cushions and thermoplastic shells, and computed tomography (CT) simulation was performed. The gross tumor volume (GTV) was delineated as a visible lesion on lung window CT images. The median GTV was 59 (range, 11−371) cm^3^. The clinical target volume (CTV) margin was set at 5–8 mm to encompass subclinical disease extension. The planning target volume (PTV) margin included the setup and internal margins that were determined by tumor motion on four-dimensional CT images. Fifteen patients (65%) were treated with carbon-ion radiotherapy of 52.8–60.0 Gy (relative biological effectiveness [RBE]) in 4 fractions and 8 (35%) were treated with carbon-ion radiotherapy of 64.0–70.4 Gy (RBE) in 16 fractions. Elective lymph node irradiation was not performed, except in 2 patients (9%) with centrally located tumors.

### Assessment and follow-up

Patients were evaluated by chest CT, brain magnetic resonance imaging with gadolinium, and (18) F-fluorodeoxyglucose positron emission tomography (FDG-PET) before commencing treatment. FDG-PET scans were performed at 60 minutes after intravenous FDG injection with a maximum activity of 400 Mbq, after fasting for 6 hours. Follow-up consisted of a clinical examination every 3 months with CT imaging. FDG-PET was performed annually after carbon-ion radiotherapy. Adverse events were evaluated according to the Common Terminology Criteria for Adverse Events, version 4.0.

### Dose-volume histogram analysis

Twenty-one patients treated with carbon-ion radiotherapy were analyzed to compare DVH parameters with photon therapy. Two patients (9%) with prophylactic irradiation to the lymph node station were excluded. Photon therapy treatment plannings, including three-dimensional conformal radiotherapy (3DCRT) and intensity-modulated radiotherapy (IMRT), by multiple field irradiation were generalized using data from patients treated with carbon-ion radiotherapy. All radiotherapy treatment plans were calculated with the same target volumes and organs at risk using the same total irradiation dose. The dose covering 2% (D2), 50% (D50), 80% (D80), 95% (D95), and 98% (D98) of the PTV, and the homogeneity index (defined as [D2 –D98] / D50), were calculated. The normal lung volume receiving 5 Gy (V5), 10 Gy (V10), 20 Gy (V20), 30 Gy (V30), 40 Gy (V40), and 50 Gy (V50), and the mean lung dose were also calcucated.

### Statistical analyses

Local control, progression-free survival (PFS), and OS rates were calculated using the Kaplan-Meier method, and statistical differences were determined using the log-rank test. Differences between two groups were compared using a Student’s t-test. All statistical analyses were performed using SPSS, software version 23.0 (IBM Corp., Armonk, NY, USA). A *P* < 0.05 was considered statistically significant.

## Results

### Clinical outcomes

**Fig 1:**
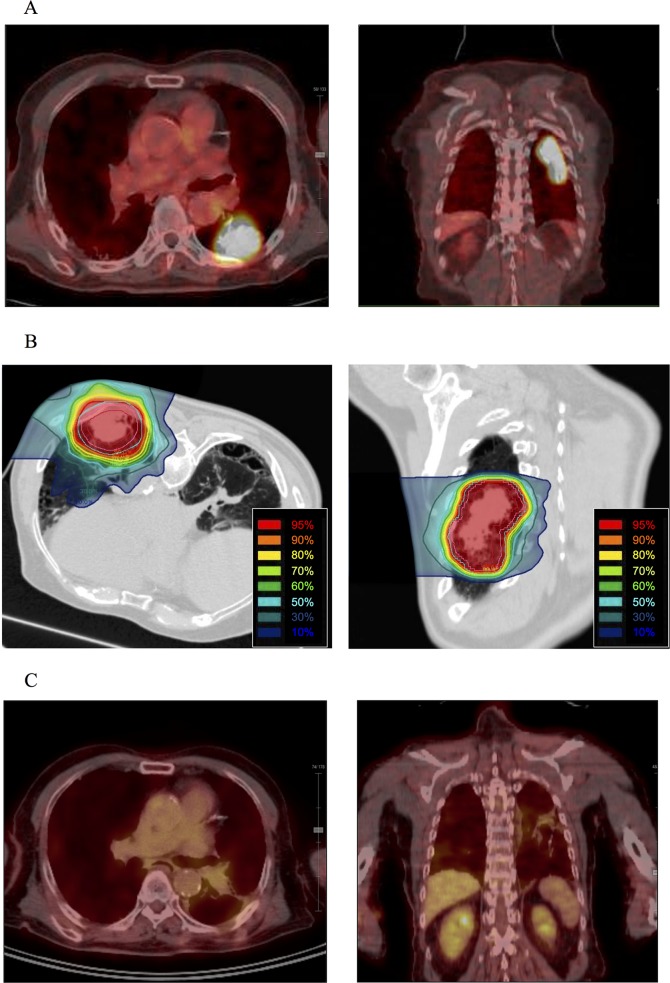
A representative case treated with carbon-ion radiotherapy. (A) A 91-year-old man with T3N0M0 lung squamous cell carcinoma. A rapidly growing tumor was 73 mm in diameter. (18) F-Fluorodeoxyglucose positron emission tomography (FDG-PET) images revealed that the tumoral maximum standardized uptake value was 12.1 and tumor to normal tissue (T/N) ratio was 3.8. The patient was considered inoperable because of his age and chronic obstructive pulmonary disease. (B) Carbon-ion radiotherapy was performed using a respiratory-gated technique with 60 Gy (relative biological effectiveness) in 4 fractions. The GTV, CTV, and PTV are represented by the red, cyan, and magenta lines, respectively. (C) Following treatment, asymptomatic grade 1 radiation pneumonitis developed but additional treatment was not required. Two years after carbon-ion radiotherapy, FDG-PET images demonstrated reductions in the maximum standardized uptake value from 12.1 to 2.5 and in the T/N ratio from 3.8 to 1.0. Distant or lymph node metastases were not detected. The patient was still alive without disease progression at 3 years after carbon-ion radiotherapy.

**Fig 2:**
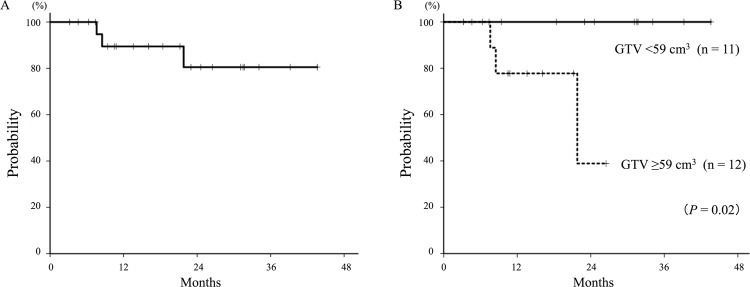
Local control curves of T2b–4N0M0 non-small cell lung cancer patients treated with carbon-ion radiotherapy. (A) The 2-year local control rate for all patients (n = 23) was 81%. (B) The 2-year local control rates of patients with a gross tumor volume (GTV) of <59 cm^3^ (n = 11) or ≥59 cm^3^ (n = 12) were 100% and 39%, respectively (*P* = 0.02).

The median follow-up of the surviving patients was 25 (range, 4–54) months. All 23 patients completed carbon-ion radiotherapy, and a representative case is shown in **Fig 1A–1C**. Three patients (13%) experienced local recurrence, and the 2-year local control rate was 81% (**Fig 2A**). One patient with local recurrence underwent salvage surgery and survived 32 months after resection without the development of severe postoperative adverse events. The other 2 patients with local recurrence underwent carbon-ion radiotherapy or chemotherapy and died of disease progression at 10 and 11 months after each treatment, respectively. The 2-year local control rates for T2b (n = 7) and T3–4 (n = 16) were 80% and 83%, respectively, although the difference was not statistically significant (*P* = 0.76). The 2-year local control rates of patients with a GTV of <59 cm^3^ (n = 11) or ≥59 cm^3^ (n = 12) were 100% and 39%, respectively (*P* = 0.02; **Fig 2B**). Other clinical characteristics, such as tumor length, pathology, age, operability, and radiation dose, were not significant factors for local control.

Two mediastinal lymph node recurrences, 2 malignant pleural effusions, and 5 distant metastases (bone [n = 2], brain [n = 2], and multiple [n = 1] metastases) were detected during follow-up. The 2-year PFS rate for all 23 patients was 37%. For the 2 patients with lymph node recurrences, additional carbon-ion radiotherapy was performed, and the patients survived without disease progression at 8 and 25 months after irradiation, respectively. SBRT was performed for the 2 patients with brain metastases. One patient survived without disease progression at 19 months after SBRT, and the other patient died of the disease at 20 months after SBRT. Five patients with malignant pleural effusions or distant metastases underwent chemotherapy or received best supportive care, according to the patient’s conditions. The 2-year PFS rates of T2b (n = 7) and T3–4 (n = 16) were 51% and 34%, respectively, with T2b patients exhibiting a trend towards a better PFS (*P* = 0.09). Other clinical and tumor characteristics were not significant factors for PFS.

**Fig 3:**
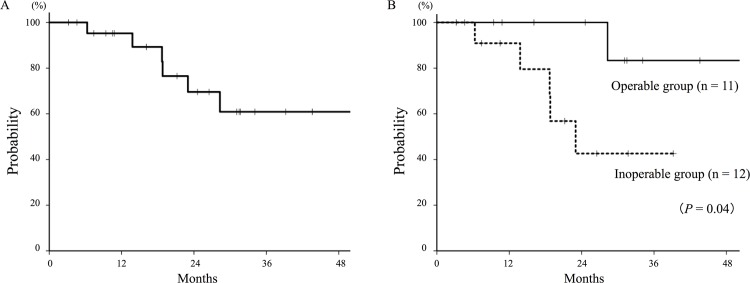
Overall survival curves of T2b–4N0M0 non-small cell lung cancer patients treated with carbon-ion radiotherapy. (A) The 2-year overall survival rate for all patients (n = 23) was 70%. (B) The 2-year overall survival rates of patients in the operable (n = 11) and inoperable (n = 12) groups were 100% and 43%, respectively (*P* = 0.04).

During follow-up, 5 patients died of lung cancer, and 1 died of intercurrent disease. The 2-year OS rate for all patients was 70% (**Fig 3A**). Operable patients (n = 11) had a significantly higher OS rate compared with inoperable patients (n = 12), with 2-year OS rates of 100% and 43%, respectively (*P* = 0.04, **Fig 3B**). The 2-year OS rates for T2b (n = 7) and T3−4 (n = 16) were 100% and 50%, respectively, although the difference was not statistically significant (*P* = 0.13). Other clinical and tumor characteristics were not significant factors for OS.

### Adverse events

The adverse events in patients treated with carbon-ion radiotherapy are summarized in **Table 2**. Although grade 1 radiation pneumonitis was common, there were no grade ≥2 cases. One patient experienced grade 2 chest wall pain, and 2 developed grade 2 dermatitis radiation, which were immediately improved by conservative treatments. No patients experienced grade ≥3 adverse events.

**Table 2:** Adverse events in patients (n = 23) treated with carbon-ion radiotherapy.

Adverse Event	Grade 1	Grade 2	Grade 3	Grade 4
Radiation pneumonitis	21 (91%)	0 (0%)	0 (0%)	0 (0%)
Dermatitis radiation	17 (74%)	2 (9%)	0 (0%)	0 (0%)
Chest wall pain	1 (4%)	1 (4%)	0 (0%)	0 (0%)
Esophagitis	2 (9%)	0 (0%)	0 (0%)	0 (0%)

### Comparative analysis of dose-volume histogram parameters

**Fig 4:**
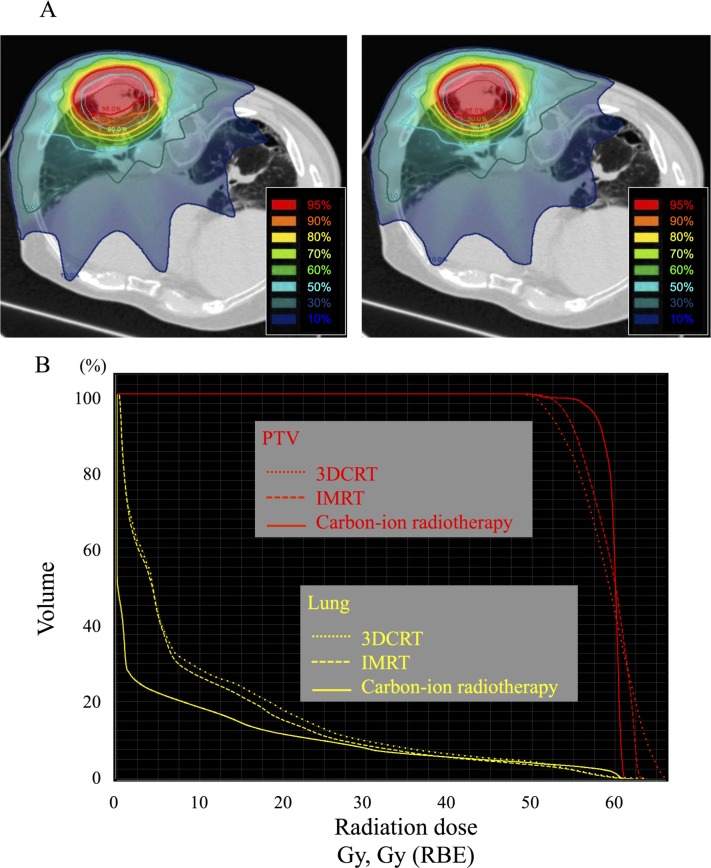
A comparison of dose distribution between carbon-ion radiotherapy and photon therapies in a representative case. (A) Left: The dose distribution of three-dimensional radiotherapy (3DCRT). Right: The dose distribution of intensity-modulated radiotherapy (IMRT). The irradiated volume of the carbon-ion radiotherapy (See Fig 1B) was smaller compared with those of the photon therapies. (B) Dose-volume histograms of the planning target volume (PTV; red) and lung (yellow) for the carbon-ion radiotherapy (solid line), IMRT (dashed line), and 3DCRT (dotted line). RBE, relative biological effectiveness.

**Fig 5:**
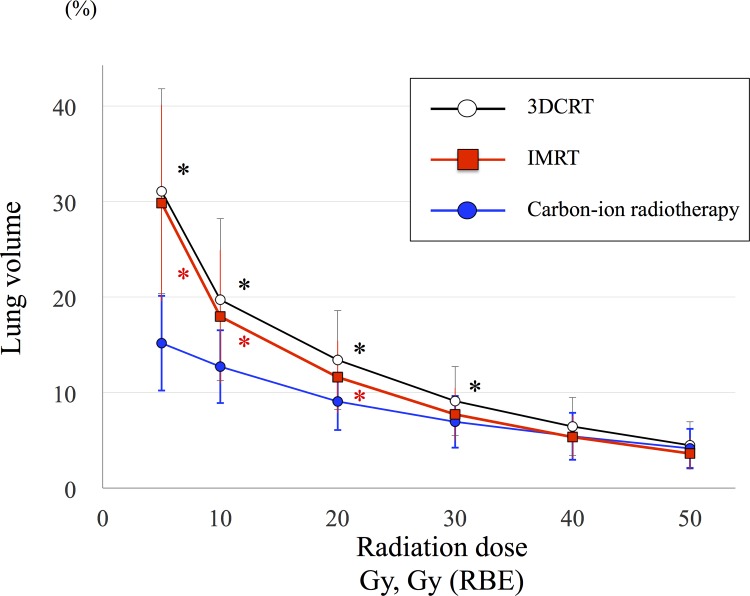
Comparisons of lung dose-volume between carbon-ion radiotherapy and photon therapies. Carbon-ion radiotherapy is represented by the blue line, three-dimensional radiotherapy (3DCRT) by the black line, and intensity-modulated radiotherapy (IMRT) by the red line. 3DCRT had significantly higher irradiated lung volumes of V5, V10, V20, and V30 compared with the carbon-ion radiotherapy (Black asterisk). IMRT had significantly higher irradiated lung volumes of V5, V10, and V20 compared with the carbon-ion radiotherapy (Red asterisk). Asterisks (*) represent *P* < 0.05. RBE, relative biological effectiveness; Vx, normal lung volume receiving X Gy.

**Fig 4A and 4B** shows the dose distribution and DVH parameters of a representative case for carbon-ion radiotherapy and photon therapies, including 3DCRT and IMRT. The average lung volumes irradiated with carbon-ion radiotherapy and photon therapies are displayed in **Fig 5**. Carbon-ion radiotherapy was associated with a significantly smaller irradiated lung volumes of V5, V10, V20, and V30 compared with 3DCRT or IMRT (*P* < 0.05). Similarly, the mean lung dose of carbon-ion radiotherapy (5.1 ± 1.8 Gy [RBE]) was significantly less than that of 3DCRT (.0 ± 2.8 Gy; *P* < 0.001) or IMRT (7.2 ± 2.2 Gy [RBE]; *P* < 0.001). A comparison of PTV coverage is displayed in **Table 3**. The near minimum dose covering the PTV (D98) of carbon-ion radiotherapy was significantly higher compared with those of 3DCRT or IMRT. Moreover, carbon-ion radiotherapy had a significantly lower D2, near maximum dose, compared with the photon therapies. Therefore, the carbon-ion radiotherapy had half the homogeneity index of the photon therapies, revealing that it provided a more homogeneous dose distribution to the tumor. DVH analysis suggested that carbon-ion radiotherapy could spare the dose to normal lung tissue and focus on the tumor.

**Table 3:** Comparison of planning target volume (PTV) parameters between carbon-ion radiotherapy and photon therapies, including three-dimensional radiotherapy (3DCRT) or intensisty-modulated radiotherapy (IMRT).

	Carbon-Ion Radiotherapy	3DCRT		IMRT	
	(Mean ± SD)	(Mean ± SD)	*P*-value	(Mean ± SD)	*P*-value
D2	62.2 ± 2.0 Gy (RBE)	65.0 ± 3.8 Gy	0.007	64.6 ± 1.6 Gy	<0.001
D50	61.4 ± 1.8 Gy (RBE)	60.0 ± 3.1 Gy	0.154	61.3 ± 1.8 Gy	0.706
D80	60.7 ± 1.9 Gy (RBE)	57.3 ± 3.1 Gy	<0.001	58.9 ± 2.3 Gy	0.009
D95	58.8 ± 3.3 Gy (RBE)	55.0 ± 3.2 Gy	0.001	56.4 ± 2.5 Gy	0.012
D98	57.4 ± 3.9 Gy (RBE)	54.0 ± 3.3 Gy	0.005	55.0 ± 2.7 Gy	0.030
HI	0.08 ± 0.07	0.18 ± 0.04	<0.001	0.16 ± 0.05	<0.001

DX, dose covering X% of the planning target volume; RBE, relative biological effectiveness; SD, standard deviation; HI, homogeneity index; 3DCRT, three-dimensional radiotherapy; IMRT, intensity-modulated radiotherapy.

## Discussion

In the present study, we revealed the efficacy and safety of carbon-ion radiotherapy for T2b–4N0M0 NSCLC patients. The 2-year local control and OS rates were 81% and 70%, respectively, which were comparable to those reported in a previous carbon-ion radiotherapy study. Takahashi et al. [15] published a Phase I/II study of carbon-ion radiotherapy of 68–76 GyE in 16 fractions for locally advanced NSCLC patients (n = 62) with or without lymph node metastases. The 2-year local control and OS rates of the entire cohort were 93.1% and 51.9%, respectively. Of the 23 patients without lymph node metastasis, the 2-year local control and OS rates were 100% and 69.3%, respectively. Therefore, the authors concluded that the group of locally advanced NSCLC patients without lymph node metastases was a good candidate for carbon-ion radiotherapy [15]. In the present study, 3 patients (13%) developed local recurrence with a 2-year local control rate of 81%, which was less than that in the previous study. One possible cause of this discrepancy relates to the inclusion criteria because Takahashi et al. [15] excluded tumors >7 cm. By contrast, 43% of tumors were >7 cm in the present study, and patients with larger tumors had significantly poorer local control than those with smaller tumors (*P* = 0.02). Another possible reason is the difference in carbon-ion radiation doses between the studies, because the previous study used 68–76 GyE [15], whereas the majority of patients (91%) in the present study were treated with 60–64 Gy (RBE).

In the Takahashi et al. [15] study, 4 patients (6%) developed grade 2 radiation pneumonitis, and 1 (2%) developed grade 3 radiation pneumonitis. In addition, 1 patient (2%) experienced a grade 3 tracheoesophageal fistula. In the present study, the radiation pneumonitis rates were extremely low with a grade 2 toxicity of 0%. It is possible that the relatively lower radiation doses in the present study underlie the lack of severe adverse events. Moreover, our facility does not adopt elective lymph node irradiation, which results in a low rate of adverse events associated with the lungs or mediastinal organs. There was concern that an involved field without elective irradiation could increase the risk of lymph node metastases. However, pre-treatment FDG-PET imaging has been shown to detect mediastinal lymph node metastases accurately [16, 17]. Therefore, the number of isolated metastases has decreased, and elective irradiation has been accepted gradually [18, 19]. In the present study, isolated mediastinal lymph node metastases after carbon-ion radiotherapy were detected in 2 patients (9%) and additional carbon-ion radiotherapy was successful in treating these patients. Given these findings of a low rate of adverse events and lymph node metastases, we recommend an involved field approach to primary tumor irradiation without elective lymph node irradiation for T2b–4N0M0 NSCLC.

In the present study, radical treatments for disease recurrence were performed according to the patient’s conditions. For 1 patient with local recurrence, salvage surgery was performed, and the patient survived at 32 months after resection. However, non-resection treatments did not result in long-term survival. Recently, salvage surgery was reported to be feasible and safe for 12 NSCLC patients treated with carbon-ion radiotherapy [20]. Given these findings, salvage surgery for local tumor recurrence after carbon-ion radiotherapy could be a promising strategy. For 2 patients with lymph node recurrences in the present study, additional carbon-ion radiotherapy was performed, and the patients survived without disease at 8 and 25 months after irradiation. The treatment for mediastinal lymph node recurrences has not been established. Recently, SBRT for mediastinal lymph node metastases was reported to be a safe and efficacious treatment for patients without previous mediastinal radiotherapy [21]. Although chemotherapy is regarded as the first choice for recurrence, radical local treatments could be considered because patients with solitary local recurrence and lymph node metastases without distant metastases are expected to have a relatively long survival.

In the present study, operable patients had a significantly better OS rate compared with inoperable patients. A Japanese multi-institutional study of SBRT for stage I NSCLC [22] also reported that operable patients had a significantly better OS rate compared with inoperable patients. Inoperable patients frequently die of other causes, although, in the present study, only 1 patient (4%) died of intercurrent disease. Several other explanations might be associated with a higher OS rate in operable patients. When local recurrence occurs, salvage surgery is a potential radical treatment for operable patients, but not for inoperable patients [20]. Recently, molecularly targeted therapy, immunotherapy, and novel chemotherapy have been established for improving OS rates for patients with recurrent or metastatic disease [23–25], and these systematic therapies also fully benefit those patients who are operable and have a better PS. Given these findings, operable and inoperable patients should be categorized into different groups, and optimized treatment strategies should be implemented for each group.

The present study revealed that carbon-ion radiotherapy exhibited better target conformity with lower doses in the normal lung tissue than photon therapies, such as 3DCRT and IMRT, in T2b–4N0M0 NSCLC. To the best of our knowledge, this is the first study demonstrating the superior DVH parameters of this stage. Ebara et al. [26] demonstrated that carbon-ion radiotherapy had significantly better lung DVH parameters for stage I lung cancer compared with SBRT. Kubo et al. [27] evaluated the DVH parameters of stage IIIA patients with N2 lymph nodes and revealed that the dose distributions of carbon-ion radiotherapy were more favorable compared with those of photon therapy. Furthermore, they found that carbon-ion radiotherapy could decrease the lung dose-volume, as well as that of the esophagus, vertebral body, and trachea, without a reduction in tumor dose coverage [27]. Given these findings, carbon-ion radiotherapy appears to have a superior dose profile than photon therapy for several stages of lung cancer.

Conventional photon therapy has clear limitations regarding local controls for locally advanced NSCLC without lymph node metastasis. However, the clinical outcomes of hypofractionation therapy and proton therapy have recently been reported. A Phase II SBRT study [28] revealed that T2b tumors (5−7 cm, diagnosed as stage IIA), were as safely treated as stage I tumors. A retrospective study showed that SBRT for T3–T4N0M0 NSCLC was effective and feasible [29]. A study of proton therapy [30] with concurrent chemotherapy for locally advanced NSCLC reported superior OS rates without severe adverse events for stage II/III tumors. Hypofractionation photon therapy has been reported to be efficacious for locally advanced NSCLC [31, 32]. However, most of these studies were conducted in patients with stage III disease and did not include information regarding nodal status. In future trials of locally advanced NSCLC, optimal radiotherapy approaches should be considered for groups of patients with or without lymph node metastases.

This study had some limitations, such as the retrospective design, a small number of patients, and relatively short follow-up. Because of the retrospective nature, the dose fractionations were various and based on a few previous treatment regimens. Although optimal radiotherapy protocols for T2b-T4N0M0 NSCLC have not been established, the report on carbon-ion radiotherapy could be considered useful.

## Conclusions

Carbon-ion radiotherapy was safely performed for T2b–4N0M0 NSCLC. Furthermore, carbon-ion radiotherapy resulted in favorable outcomes with 2-year local control and OS rates of 81% and 70%, respectively. Operability was a significant prognostic factor for OS. DVH analysis revealed that carbon-ion radiotherapy could significantly reduce the lung dose-volume and improve tumor coverage compared with 3DCRT or IMRT. A Japanese multi-institutional study is ongoing to prospectively evaluate the efficacy and safety of carbon-ion radiotherapy for T2b–4N0M0 NSCLC.
